# Conserved *Cis*-Regulatory Modules Control Robustness in *Msx1* Expression at Single-Cell Resolution

**DOI:** 10.1093/gbe/evv179

**Published:** 2015-09-04

**Authors:** Keith W. Vance, Dan J. Woodcock, John E. Reid, Till Bretschneider, Sascha Ott, Georgy Koentges

**Affiliations:** ^1^Department of Biology and Biochemistry, University of Bath, United Kingdom; ^2^Warwick Systems Biology Centre, University of Warwick, Coventry, United Kingdom; ^3^School of Life Sciences, University of Warwick, Coventry, United Kingdom; ^4^MRC Biostatistics Unit, Robinson Way, Cambridge, United Kingdom

**Keywords:** single-cell transcription, *cis*-regulatory module, *Msx1*, promoter, robustness

## Abstract

The process of transcription is highly stochastic leading to cell-to-cell variations and noise in gene expression levels. However, key essential genes have to be precisely expressed at the correct amount and time to ensure proper cellular development and function. Studies in yeast and bacterial systems have shown that gene expression noise decreases as mean expression levels increase, a relationship that is controlled by promoter DNA sequence. However, the function of distal *cis-*regulatory modules (CRMs), an evolutionary novelty of metazoans, in controlling transcriptional robustness and variability is poorly understood. In this study, we used live cell imaging of transfected reporters combined with a mathematical modelling and statistical inference scheme to quantify the function of conserved *Msx1* CRMs and promoters in modulating single-cell real-time transcription rates in C2C12 mouse myoblasts. The results show that the mean expression–noise relationship is solely promoter controlled for this key pluripotency regulator. In addition, we demonstrate that CRMs modulate single-cell basal promoter rate distributions in a graded manner across a population of cells. This extends the rheostatic model of CRM action to provide a more detailed understanding of CRM function at single-cell resolution. We also identify a novel CRM transcriptional filter function that acts to reduce intracellular variability in transcription rates and show that this can be phylogenetically separable from rate modulating CRM activities. These results are important for understanding how the expression of key vertebrate developmental transcription factors is precisely controlled both within and between individual cells.

## Introduction

Transcription is a fundamentally stochastic process that occurs discontinuously in bursts in single cells as described by random telegraph models of gene expression (Paulsson 2005; Raser and O’Shea 2005; Cai et al. 2006; Raj et al. 2006; Pedraza and Paulsson 2008; Suter et al. 2011). Experiments in microbial systems and in *Drosophila* have revealed that stochasticity in gene expression can be actively used to generate cellular diversity of adaptive value in large cell populations (Samoilov et al. 2006; Wernet et al. 2006), whereas in vertebrates cell-to-cell variations in the expression of key developmental regulators in progenitor cells have been shown to drive cell fate choices during lineage differentiation (Chang et al. 2008; Raj and van Oudenaarden 2008). Comparative embryology and developmental genetics, on the other hand, have revealed that transcription factor and signaling networks are expressed and deployed in a highly stereotypic and exquisitely precise spatiotemporal fashion during development. Furthermore, studies in yeast have shown that the expression of essential, haplo-insufficient genes is controlled in a more precise manner when compared with nonessential genes, such as those involved in the stress response, and that noise in the expression of these dose-dependent genes appears to have been reduced by natural selection to prevent deleterious stochastic variations (Newman et al. 2006; Batada and Hurst 2007; Lehner 2008). Random fluctuations in gene expression are therefore subject to regulation and can be either beneficial or deleterious for cell function depending on the type of gene and biological process.

Noise and robustness in gene expression can be controlled at a number of different levels. A large number of studies in yeast and bacteria have demonstrated that noise in gene expression decreases as mean expression levels increase (Bar-Even et al. 2006; Newman et al. 2006), and that promoter sequence, length and chromatin structure are all major determinants of this relationship (Tirosh and Barkai 2008; Hornung et al. 2012; Carey et al. 2013). In addition, random fluctuations in gene expression can be buffered at both the RNA and protein levels (Raser and O’Shea 2005; Pedraza and Paulsson 2008; Barriere et al. 2011), whereas signaling pathways such as *Wnt* have been proposed to filter out and reduce transcriptional noise (Arias and Hayward 2006). Moreover, transcription factors do not function in isolation but work in a highly coordinated manner within gene regulatory networks (GRNs) and particular network motifs, for example, feed-forward or feedback loops, have been shown to buffer out fluctuations in gene expression (Barkai and Leibler 1997; Alon et al. 1999; Mangan and Alon 2003; Macneil and Walhout 2011).

Within GRNs key transcription factors interact with DNA sequence elements called *cis*-regulatory modules (CRMs), such as transcriptional enhancers and silencers, and promoters, to control the expression of large numbers of target genes (Jeziorska et al. 2009). Communication between CRMs and their target promoters is a key metazoan novelty in transcriptional control. CRMs may be predicted to play an important role controlling transcriptional noise as, for example, shadow enhancers in *Drosophila* have been shown to promote gene expression robustness in response to various stimuli (Boettiger and Levine 2009; Frankel et al. 2011). However, the full dynamic scope of CRM function beyond spatiotemporal control is still to be determined as the majority of studies investigating the control of transcriptional noise at single-cell resolution have been performed in bacteria and yeast. As such, the involvement of CRMs in regulating transcriptional variability has not been well explored (Macneil and Walhout 2011).

In this study, we used live cell imaging of transfected reporters and mathematical modeling to quantify the function of mouse–fugu conserved *Msx1* CRMs and promoters in regulating single-cell transcription rates in real time in C2C12 mouse mesenchymal cells. We predicted that variability in the expression of *Msx1* would be finely controlled as *Msx1* is an important regulator of pluripotency in mesenchymal stem cells, acting at a key node in a GRN, to control proximodistal branchial arch patterning and craniofacial and dorsal CNS development (Satokata and Maas 1994; Odelberg et al. 2000; Hu et al. 2008). Also, the spatial and temporal expression of *Msx1* is highly conserved across vertebrates and appears to show an ON/OFF behavior akin to ultrasensitivity (Hu et al. 2008). Our results showed that the negative correlation between transcriptional noise and mean expression is not determined by distal CRMs but is solely promoter derived for vertebrate *Msx1*. We found that *Msx1* CRMs function to modulate single-cell basal promoter rate distributions in a graded manner across a population of cells. This further refines the rheostatic model of CRM action, proposed on the basis of single time point reporter measurements, to provide a more detailed understanding of CRM function. Unexpectedly, our results also identified a novel CRM transcriptional filter, or robustness control, function that acts to reduce intracellular variability in transcription rates. By combining promoters with homologous mouse–fugu CRMs, we identified examples of conservation and divergence in CRM function and showed that CRM robustness control can be phylogenetically separable from rate modulating CRM activities. These results are important for understanding how the expression of key developmental regulators is finely controlled, which in principle will also have important ramifications for synthetic biology and gene therapy.

## Materials and Methods

### Plasmid Construction

The pGL3-vGFP3xnls Venus fluorescent protein reporter plasmid used as a backbone for all species-specific CRM-promoter reporter constructs was cloned as follows: Recombinant polymerase chain reaction (PCR) was performed to insert a triple nuclear localization sequence in frame at the 3′-end of the Venus gene from pCS2Venus. This generated a vGFP3xnls fragment flanked by *Nco*I–*Xba*I sites. The luciferase gene from pGL3-BASIC (Promega) was then excised as an *Nco*I–*Xba*I fragment and replaced with vGFP3xnls. Species-specific CRM-promoter hybrid fluorescent reporters were then generated as follows: The mouse and fugu *Msx1* promoter regions were PCR cloned as *Hin*dIII fragments from genomic DNA and inserted into pGL3-vGFP3xnls. The SV40 promoter was PCR amplified as a *Hin*dIII fragment from pGL3-PRO (Promega) and cloned into pGL3-Venus. Individual species-specific CRMs were then PCR amplified as *Xma*I–*Bgl*II fragments from genomic DNA and cloned upstream of the respective promoter Venus reporters. The fidelity of all reporter constructs was verified by sequencing. The oligonucleotides used are shown in supplementary table S1, Supplementary Material online.

### Cell Culture

C2C12 myoblast cells were grown in Dulbecco’s modified Eagle’s medium supplemented with 10% fetal bovine serum (FBS) (Growth Medium [GM]).

### Flow Cytometry

Flow cytometry-based measurement of reporter activity was performed as described in Jeziorska et al. (2012). 2 × 10^5^ cells per well were seeded in six-well plates. The next day cells were transfected with 1 µg of pGL3-vGFP3xnls reporter plasmid and 250 ng pCMV-mCherry expression vector using lipofectamine 2000 (Invitrogen). pCMV-mCherry contains the cytomegalovirus promoter driving the expression of a mCherry reporter and was used to control for transfection efficiency. After 42–46 h cells were washed twice with phosphate-buffered saline (PBS), trypsinized, and harvested in 1 ml GM. GM was removed, cells were then washed twice with PBS, resuspended in 800 μl Cell Fix (BD Biosciences), and incubated overnight at 4 °C in the dark. To activate *wnt* signaling, 40 mM LiCl was added to each well 20 h after transfection.

To measure MSX1 protein levels, cells were pelleted, washed with PBS, and then permeabilized using 0.1% Triton X-100 in PBS for 15 min at room temperature. Cells were subsequently washed with PBS and blocked using 20% FBS in PBS for 1 h at room temperature. Cells were then incubated overnight at 4 °C with 1:200 dilution anti-Msx1 (Clone 4FII; Abcam) mouse monoclonal antibody, washed three times with 5% FBS in PBS, and incubated for 1 h with 1:200 dilution goat anti-mouse AlexaFluor647 antibody (Invitrogen). Cells were washed again with PBS, resuspended in 500 μl Cell Fix, and analyzed on a BD Influx Cell Sorter. Venus was excited using the blue laser (488 nm) and measured with the 530/40 bandpass (BP) filter set, mCherry was excited using the yellow laser (561 nm) and measured using the 593/40 BP filter whereas Alexa647 was excited with the red laser (642) and measured using the 670/30 BP filter set.

### Real-Time Imaging

1.25 × 10^4^ cells per well were seeded in 0.17-mm glass bottom 96-well plates (MatriCal). The next day cells were treated with Hoechst 33342 (Invitrogen) to label individual nuclei. To do this, GM was removed, cells were washed twice with PBS, and then incubated with 400 ng/ml Hoechst in GM at 37°C for 30 min. Cells were then washed twice with PBS and GM without phenol red was added back to the well. Cells were then transfected with 200 ng reporter plasmid using Lipofectamine 2000 (Invitrogen) according to the manufacturer’s instructions. The 96-well plate was subsequently transferred to a Cellomics KineticScan KSR machine. The KSR contains a humidified incubator, an inverted fluorescent Zeiss microscope with a high resolution CCD camera, and an integrated computer system. Real-time images were generated with a 10× magnification, 0.4 numerical aperture objective using both the Hoechst and GFP filter sets. The Hoechst channel was used to focus and we acquired images every 30 min for 48 h. Cells were segmented, tracked, and fluorescence levels extracted using custom software. The autofluorescence GFP value of an untransfected cell reached a maximum of 7,000 units. To remove background, we set a preselection threshold value of 8,000 fluorescent units in the GFP channel and measured the response profiles for cells which have at least ten measurement points above this value. We synchronized fluorescence response profiles in silico to the point of cell division as determined by a rise and then postmitotic drop in Hoechst staining. This enabled us to restrict fluorescent reporter measurements to individual cell generations. We previously calculated that 15 or more cells per experiment were required for robust transcription rate estimates (Woodcock et al. 2013) and therefore randomly selected 30–35 fluorescent onset curves for each reporter construct.

### Extraction of Single-Cell Fluorescence Time-Course Data

Segmentation based on the nuclear stain (Hoechst) was performed using standard routines built into KineticScan V2.2.0.1 (BUILD 27) software (Thermo Scientific) and data were exported to a Microsoft Access database. For subsequent tracking we extracted cell positions and fluorescence intensities using custom plugins for ImageJ software (http://rsb.info.nih.gov/ij) employing Jackcess, a java library for reading from and writing to Access databases (http://jackcess.sourceforge.net/). We have developed novel routines for cell tracking written in C based on a statistical scoring algorithm which accounts for dense cell cultures at 10× or 20× resolution and significant movement, that is, translocation of more than a cell diameter between subsequent frames. Confidence scores are computed taking into account distances to neighboring cells, extent of cell movement, and alternative cell-to-cell assignments. We have been able to automatically define up to two cell divisions in one time-course by using variations in Hoechst staining intensity between cells and a characteristic drop in Hoechst intensity after cell division. The software is described in Downey et al. (2011). Single-cell trajectories for all *Msx1* promoter containing reporters are shown in supplementary figure S1, Supplementary Material online.

### Computational Genomics

We implemented a computational approach analogous to the one described in Picot et al. (2010) to identify conserved regions.

### Hierarchical Model of Transcriptional Dynamics

The model is described in detail in Woodcock et al. (2013). This method incorporates both intrinsic and extrinsic noise into the estimation (Finkenstadt et al. 2013) and has been used to enable estimation of transcription rates when the plasmid copy number is unknown (Woodcock et al. 2013). It consists of two layers: The single-cell layer and the population layer. The single-cell layer takes the form of a pair of stochastic differential equations describing how the mRNA and protein level change in time, and an equation which describes the measurement process. These equations are

dM(t)=(cτ(t)−δMM(t))dt+cτ(t)−δMM(t)dW(t),dP(t)=(αM(t)−δPP(t))dt+αM(t)−δPP(t)dW(t),F(t)=κP(t)+εσ2,

where *c* is the copy number; *τ*(*t*) is the transcription rate at time *t*; *δ_M_* and *δ_P_* are the mRNA and protein degradation rates, respectively; *α* is the translation rate; κ is the measured fluorescence per protein; and $\varepsilon_{\sigma^{2}}$ is normally distributed measurement noise, with variance *σ*^2^. *M*(*t*), *P*(*t*) and *F*(*t*) are, respectively, the mRNA, protein and fluorescence levels at time *t* and *dW*(*t*) denotes a Weiner process. In this model, *τ*(*t*) can take one of two values depending if the gene is actively transcribing, namely *τ*_0_ when transcription is only occurring at a basal level and *τ*_1_ in the active state. We also need to estimate the time when transcription changes between these states, which we refer to as the switch time, *T*. These equations capture the difficulty in estimating transcription rate when the copy number is unknown, as their respective parameters only appear as a product and so they would not normally be identifiable in a conventional estimation procedure. This is because the estimated values could trade-off against one another, meaning there would be an infinite number of possible combinations of *τ*(*t*) and *c* that would be equally valid.

In conventional parameter estimation methods, each estimate for each cell in the sample would be undertaken separately and the results then amalgamated and analyzed as a group. In our estimation scheme, we follow Finkenstadt et al. (2013) and make the assumption that each individual rate, and therefore each parameter, comes from a distribution over the population. Collectively, these distributions are referred to as the population layer. These are then estimated concurrently with the parameters for all of the time series simultaneously. To do this, we employ a Markov chain Monte Carlo (MCMC) estimation procedure which is highlighted in figure 2. This involves iteratively updating the parameter and distribution estimates by evaluating their ability to describe the data using a likelihood function derived from the single-cell layer and the probability density functions comprising the population layer. As such, the parameter estimates feed into the population distribution estimates, which themselves feed into the parameter estimates and so on. This cyclical information transfer allows a parameter estimate to “borrow strength” from the other estimates of the same parameter, leading to a more robust estimation (Finkenstadt et al. 2013).

It is this principle that we exploit to tease apart the copy number and transcription rate. Crucially, if we assume a distribution over the transcription rate as well as a distribution over the copy number then this constrains the estimates of *c* and *τ*(*t*) to be similar to those of the rest of the population; the degree of similarity is encapsulated in the population distributions. There are still an infinite number of possible combinations, but because of the population layer we can now assign a probability to these and so as the MCMC procedure iteratively updates the parameter estimates, they will begin to coalesce into their respective distributions. As this continues, the distributions become tighter and so the number of viable combinations diminishes. Eventually the estimates converge on the parameter values which best explain the data, while accounting for the similarity constraints imposed by their respective distributions.

One caveat in this approach is that the transcription rate and copy number values the MCMC method will estimate will be somewhat arbitrary as there is no way of discerning the correct absolute value for either parameter. However, as there is no reason to think that the distribution of plasmid copies entering the cell would be different between the different constructs, we can assume the same copy number distribution for each of the constructs. This means that if we estimate the distributions and parameter estimates for the entire data set at the same time, all of the transcription rates in each of the constructs will be estimated relative to the same copy number distribution. This will still not allow us to estimate the absolute transcription rate values, but they are estimated proportional to the same copy number distribution and so can be compared with each other.

## Results

### Msx1 Promoter and Comparative CRM Identification

**Fig. 1.—:**
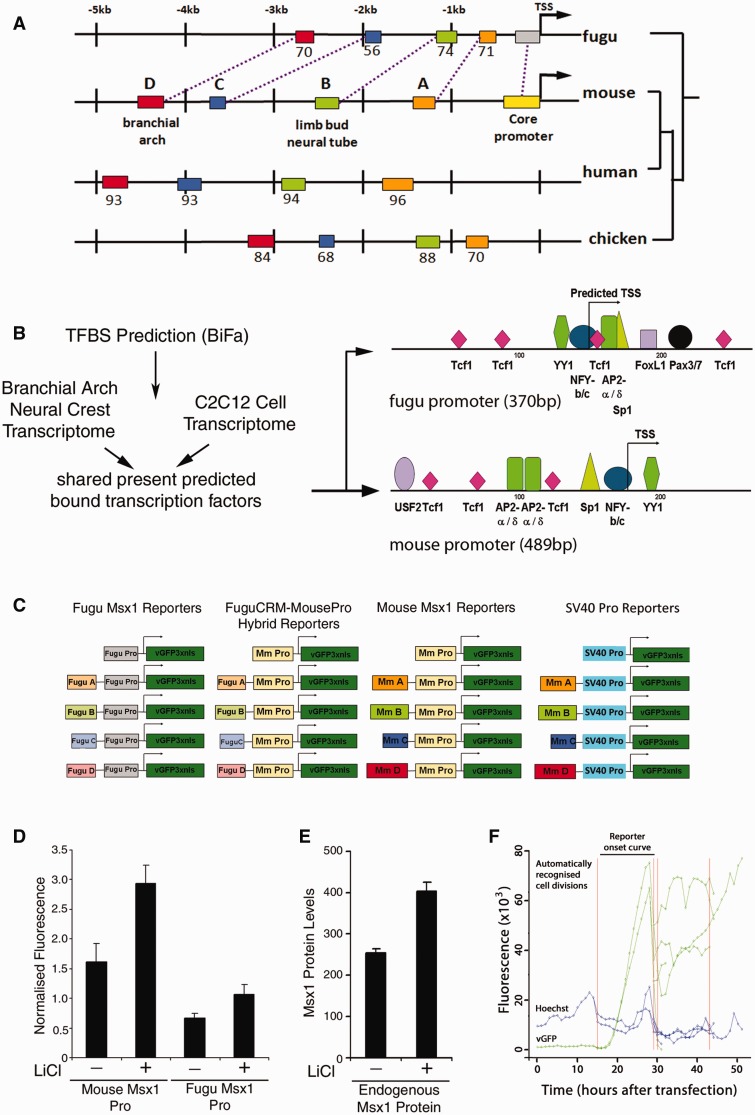
*Msx1* CRM and promoter identification. (*A*) Genomic organization of potential CRMs and promoters for mouse, human, chicken, and fugu *Msx1* based on sequence conservation. The fugu *Msx1* ortholog displays a similar configuration to that of other vertebrates. Colored boxes indicate putative regulatory regions, numbers represent percent sequence similarity to mouse. (*B*) Experimental and computational pipeline for fugu *Msx1* basal promoter identification. A conserved transcription factor promoter binding site configuration is displayed. (*C*) Single- and hybrid-species *Msx1* CRM-promoter reporter constructs. (*D*) Mouse *Msx1* basal promoter has a higher activity than the orthologous fugu region. (*E*) Endogenous MSX1 and both the mouse and fugu *Msx1* promoter reporters (shown in D) respond similarly at the quantitative level to the same exogenous stimulus. After transfection C2C12 cells were grown for 20 h and then incubated for a further 24 h with or without 40 mM LiCl. Flow cytometry was used to measure endogenous MSX1 protein and reporter fluorescence in 10,000 cells. Average *Msx1* promoter reporter fluorescence was background corrected, then normalized to the activity of a cotransfected CMV-mCherry reporter construct in each cell. (*F*) Simultaneous acquisition of fluorescent reporter and Hoechst intensity time course measurements. The SV40 Pro-vGFP reporter construct was transfected into Hoechst labeled C2C12 cells. Images were acquired at 30-min intervals after transfection and fluorescent and Hoechst intensities were extracted. Cell divisions were automatically recognized using the Hoechst channel allowing for reporter onset curves to be restricted to a single cell cycle.

We recently developed and validated a new measurement and mathematical modeling pipeline to estimate copy number-independent single-cell transcription rates using transiently transfected fluorescent reporter time courses (Downey et al. 2011; Woodcock et al. 2013). We now extend this pipeline to analyze the function of individual CRMs and promoters in controlling variability and robustness in *Msx1* expression in real time in C2C12 mesenchymal cells. The *Msx1* gene has a relatively simple *cis*-regulatory organization as a 5-kb DNA genomic fragment located immediately upstream of mouse *Msx1* can fully replicate endogenous *Msx1* expression in transgenic mice (MacKenzie et al. 1997). Using computational genomics, we identified four putative CRMs within this region, similar to the blocks of sequence conservation discovered by Miller et al. (2007), that are conserved in position and sequence between mouse and fugu (fig. 1*A*). Furthermore, we identified only one copy of *Msx1* in teleosts with a complete complement of CRMs homologous to those in mammals despite a separate round of whole-genome duplications within actinopterygians (see supplementary text, Supplementary Material online). Among these CRMs, mouse CRMB and D have previously been identified as limb bud and branchial arch enhancers (MacKenzie et al. 1997), whereas CRMA and C are uncharacterized. The mouse *Msx1* minimal promoter has previously been defined in transgenic mice and C2C12 cells in culture (Takahashi et al. 1997) and we used a custom discovery tool (Granier et al. 2011; Jeziorska et al. 2012) (see also supplementary text, Supplementary Material online) to identify the orthologous promoter region in fugu on the basis of a conserved transcription factor binding site configuration (fig. 1*B*).

### Quantitative Validation of a Reporter Assay for Studying *Msx1* Promoter–CRM Communication

*Msx1* spatiotemporal expression is highly conserved across vertebrates. However, branchial arches develop faster and morphogenetic fields are much smaller in teleosts than in mouse. This similarity and difference provide an attractive biological rationale for a quantitative functional comparison of homologous promoter and CRMs from mouse and fugu *Msx1*. To do this, we first tested our experimental system using flow cytometry to compare the activity of transfected mouse and fugu promoters with endogenous MSX1 protein levels in mouse C2C12 mesenchymal cells, an established cell line to study *Msx1* transcriptional control and its associated regulatory functions (Takahashi et al. 1997; Shetty et al. 1999; Lee et al. 2004; Wang et al. 2011; Wang and Abate-Shen 2012). This revealed that mouse basal *Msx1* promoter activity is higher compared with that of the homologous fugu promoter region (fig. 1*D*). We then treated cells with LiCl to activate the *wnt* signaling pathway, as *Msx1* is a known *wnt* target (Willert et al. 2002; Miller et al. 2007), and observed a 1.6-fold mean increase in endogenous MSX1 (fig. 1*E*) and a concomitant increase of 1.6-fold for the transfected fugu and 1.8-fold for the mouse promoter constructs (fig. 1*D*) in response to LiCl. This is within the expected range of responses to *Wnt* signaling described previously generically as well as for *Msx1* (Willert et al. 2002; Goentoro and Kirschner 2009) suggesting that transient transfection does not bias the quantitative output significantly in our system. Furthermore, the conservation of quantitative responses among orthologous *Msx1* promoters to the same input confirms our selection of the correct fugu *Msx1* promoter region.

### Estimation of Real-Time Single-Cell Transcription Rates Using Live Cell Imaging and Mathematical Modeling

To estimate copy number-independent transcription rates, we transiently transfected C2C12 cells with reporter constructs containing both inter- and same-species CRM and promoter *Msx1* components (fig. 1*C*) and performed live cell imaging to generate fluorescent onset curves for approximately 30–35 randomly picked cells for each construct (supplementary fig. S1, Supplementary Material online). We used a nuclear localized Venus fluorescent protein reporter in an effort to restrict measurement errors caused by cell size- or shape-dependent variations. We truncated the trajectories of the time courses when the maximum fluorescence was reached as our previous work showed that reporter signal is diluted and distributed equally into daughter cells after cell division (Downey et al. 2011). This enabled us to restrict fluorescent measurements to a single-cell cycle (fig. 1*F*) and minimize inaccuracies in transcription rate estimations caused by nuclear envelope breakdown and segregation of reporter plasmids after division.

**Fig. 2.—:**
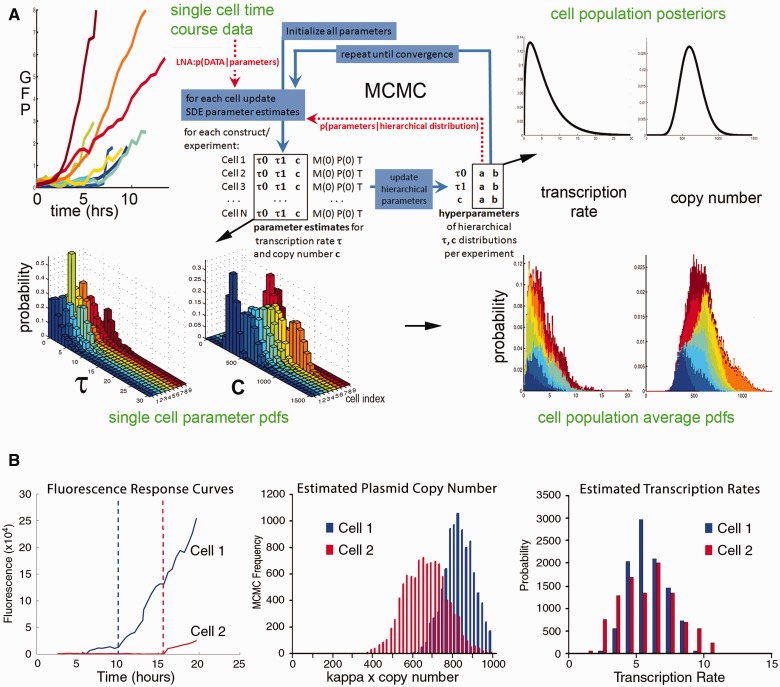
Estimation of copy number-independent transcription rates using a hierarchical model of transcriptional dynamics. (*A*) We simultaneously estimated probability density functions (pdfs) of the copy number, *c,* and transcription rate *τ1* in arbitrary units (AU) for each single-cell time course. M(0) is initial mRNA value, P(0) is the initial protein value, T is the switch time, and a and b are the parameters of the hierarchical distributions. (*B*) The fluorescence profiles of two randomly picked cells of a given experiment differ significantly (left panel). Stippled lines indicate the switch times for each individual cell. This represents the time point where transcription switches from the basal rate to the active rate. Although the calculated pdf for copy number differs between these cells (middle), the pdfs of transcription rates are very similar (right). The hierarchical estimation procedure is used to confirm the form and robustness of these “stack” plots and shows how likely a given transcription rate (in AU) is within single cells across experiments. These pdfs permit an unbiased, copy number-independent transcription rate assessment across all single cells in all experiments. In the ensuing figures, the probability densities of the stack plots showing transcription rate distributions are encoded as color intensities of heat maps along the Y-axis.

We estimated the parameters of our hierarchical model of transcriptional dynamics for single cells across all constructs using our previously described methodology developed in Woodcock et al. (2013). This inference scheme allows the estimation of all the rates in a population of cells simultaneously, which enables population information to be used in the single-cell inference and vice versa. Cyclical information transfer between the population and single cell levels guides the inference toward combinations of rates which are consistent with the rest of the population, allowing us to distinguish between the contribution of plasmid copy number and transcription rate to overall expression levels in a population of cells containing variable plasmid copy number (fig. 2*A*). Although we do not identify absolute values for the per-copy transcription rate, we are able to estimate the ratio of the per-copy transcription rate between different constructs and cells. Moreover, we have shown using synthetic data that the value of the plasmid copy number has no effect on the ability of the algorithm to correctly estimate transcription rates (Woodcock et al. 2013). The model can thus explain, for example, divergent fluorescent trajectories between two cells of the same experiment (fig. 2*B*, left) as a result of copy number (fig. 2*B*, center) rather than transcription rates (fig. 2*B*, right). For this reason, we can analyze the contributions of individual promoters and CRMs by comparing entire transcription rate distributions across all experiments. Our transient transfection approach therefore complements studies using integrated reporters in stable cell lines where the genomic location of reporter integration is known to affect the timing and size of transcriptional bursts (Becskei et al. 2005; Raj et al. 2006; Skupsky et al. 2010).

### Basal Promoters Display Dynamic Intra- and Intercellular Transcription Rate Ranges

**Fig. 3.—:**
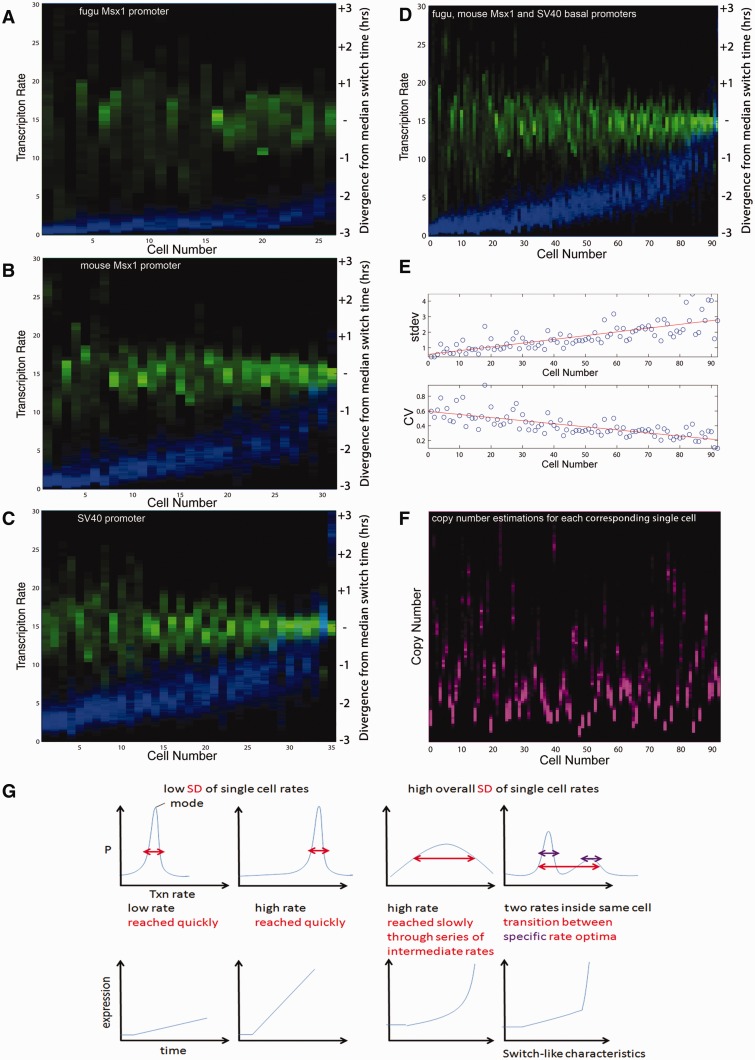
Basal promoters display intra-and intercellular transcription rate ranges. Transcription rate and switch time pdfs for the fugu *Msx1* promoter (*A*), mouse *Msx1* promoter (*B*), the *SV40* promoter (*C*), and all cells carrying basal promoter constructs (*D*). Transcription rates, plotted as heat maps at single-cell resolution, are shown in blue. Each column represents a single cell, sorted by average intracellular transcription rate on the *y-*axis (in comparable AUs), the color intensity in the heat map indicates the probability of a particular rate. For each single cell, the switch times (in green) are centered around the median switch time to allow comparisons across cells irrespective of the absolute activation time. This display format also applies to figures 4–7. (*E*) The width of the intracellular rate spectrum (measured as SD) increases with the average rate, but the CV (=SD/mean rate of each single cell) decreases. The corresponding line of best fit is shown in red. (*F*) The estimated DNA template copy numbers (pink) for all cells in (*D*) with mean transcription rates sorted in ascending order illustrate that there is no relationship between transcription rate and copy number seen as a result of our estimation process of these variables. (*G*) We observe four different classes of promoter transcription rate behaviors within single-cell time courses. The top row shows a representative rate distribution arising from each of the four classes and the bottom row shows the corresponding time series.

Studies in yeast and bacteria have shown that basal promoters are important determinants of the inverse relationship between mean expression and noise (Tirosh and Barkai 2008; Hornung et al. 2012; Carey et al. 2013). We therefore analyzed the real-time activity of the orthologous mouse and fugu *Msx1* promoters at single-cell resolution to test whether this relationship also holds true for metazoans. To examine both the rate distributions within a given cell and the population-wide distributions of average single-cell transcription rates, we displayed the calculated transcription rate distributions for each cell along the *y*-axis and sorted each individual cell along the *x*-axis according to ascending average transcription rates for all graphs. This revealed single-cell transcription rate distributions with promoter-specific ranges and showed that the corresponding range of switch times derived from the posterior distribution narrows with increased transcription rate (fig. 3*A*–*C*). This suggests that the progression from the basal to the active state occurs more rapidly in those cells with a high transcription rate. The data also showed that the basal mouse *Msx1* promoter has a 2.3-fold higher mean single-cell transcription rate compared with the orthologous fugu sequence (table 1). This is consistent with the population average measurements in figure 1*D* determined using flow cytometry, further validating our single cell approach.

**Table 1:** Population Level Statistics Showing Mean and Stdev of Single Cell Mean Transcription Rate

	Mean	Stdev
Basal Promoters		
Fugu Msx1 Pro	2.35	1.73
Mm Msx1 Pro	5.38	3.64
SV40 Pro	11.65	9.23
MouseCRM-MousePRO		
MmMsx1PRO	5.38	3.64
MmCRMA	4.53	3.65
MmCRMB	7.67	6.28
MmCRMC	11.38	9.4
MmCRMD	9.56	8.36
FuguCRM-MousePRO		
MmMsx1PRO	5.38	3.64
FuguCRMA	9.68	9.35
FuguCRMB	8.49	8.02
FuguCRMC	6.17	5.32
FuguCRMD	14.02	10.7
FuguCRM-FuguPRO		
FuguMsx1PRO	2.35	1.73
FuguCRMA	3.04	2.29
FuguCRMB	2.61	2.02
FuguCRMC	6.17	2.48
FuguCRMD	5.12	4.5
MouseCRM-SV40PRO		
SV40PRO	11.65	9.23
MmCRMA	2.72	2.08
MmCRMB	17.64	16.64
MmCRMC	13.85	12.41
MmCRMD	12.17	10.18

We assembled all basal promoter construct single-cell rates to investigate the relationship between mean transcription rate and noise. This demonstrated that the range of transcription rates inside each single cell (measured as SD) increases in correlation with the average rate (fig. 3*D* and *E*). However, the average intracellular transcription rate increases faster than the corresponding SD of rates inside each cell resulting in a decrease in the coefficient of variation (CV) per single cell with increasing average rate. Intracellular variation in transcription rates would in our context be understood as 1/CV (the signal-to-noise ratio [SNR] in control theory) of single-cell transcription rate distributions implying that promoters on their own already display an (average-rate dependent) robustness behavior, consistent with previous analyses examining the relationship between mean rate and noise in yeast and bacteria (Tirosh and Barkai 2008; Hornung et al. 2012; Carey et al. 2013). One trivial explanation could be that more transfected templates in a given cell would, when averaged over time across a single cell, lead to an overall higher level of reporter fluorescence and underlying transcription rate. Importantly, we showed that this is not the case here as the estimated template copy numbers of the cells in figure 3*F* (*y*-axis) do not correlate with increased average transcription rates (fig. 3*D*).

Our analysis revealed four different dynamic promoter behaviors within single cells (fig. 3*G*). Cells can acquire either a high or a low average rate very quickly (identified by a low SD and narrow spread of switch times), the range of rates can be traversed slowly (with high SD) or there might be more than one transcription rate optimum per cell, hinting at a variety of preferred promoter states.

### *Msx1* CRMs Modulate Single-Cell Promoter Transcription Rate Distributions in a Graded Manner

**Fig. 4.—:**
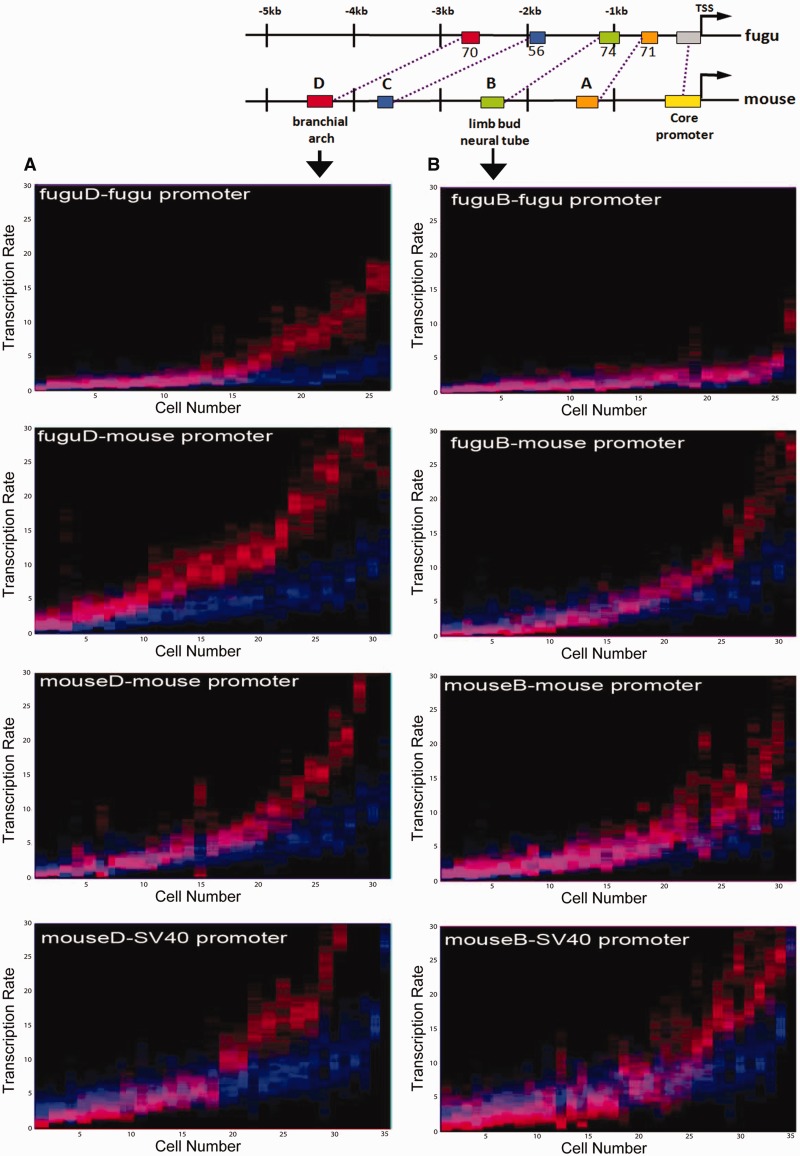
Classical *Msx1* enhancers increase the number of cells with a higher mean transcription rate in a graded manner. Basal promoter transcription rate probabilities are in blue, whereas those of constructs carrying the CRMD (*A*) and CRMB (*B*) are plotted in red. Overlap in pink indicates rates where enhancer action does not differ from the basal promoter alone. Note that even under the action of these classical enhancers transcription rate distributions are wide within single cells and many show more than one optimal set of rates, irrespective of copy number. The entire rate distribution (and not a single rate) is modified by enhancer action.

It has been proposed that CRMs use either a rheostatic or binary mode of action to modulate fixed promoter rates (for a review see Jeziorska et al. [2009]). However, single time point population measurements are unable to distinguish between these two models. To generate a more detailed understanding of CRM function, we assayed both for the ability of *Msx1* CRMs to modulate real-time promoter rate distributions in single cells, and also for conservation and divergence of function between mouse and fugu CRMs. As *Msx1* CRMD and CRMB are known to function as classical enhancers both in vivo and in C2C12 cells (MacKenzie et al. 1997; Woodcock et al. 2013), we compared the transcription rates of CRMD and CRMB containing reporters with the respective promoter-alone constructs within a population of cells. We first noted that fugu and mouse CRMD and CRMB increase mean promoter single-cell transcription rates when analyzed across a population of cells (table 1). The transcription rate distributions of CRMD and CRMB (in red) show that both fugu and mouse CRMD (fig. 4*A*) and CRMB (fig. 4*B*) shift the respective basal promoter rate distributions (in blue) upwards: more cells within a population display a higher average rate. Closer inspection of the transcription rate distributions in figure 4*A*, however, also suggests that mouse CRMD (red) has the capacity to function as either an activator or a repressor of the SV40 promoter in single cells depending on basal promoter rates (blue). The population of cells with a lower mean promoter transcription rate is repressed by mouse CRMD, whereas the population with a higher mean transcription rate is activated. This leads to a bimodal rate distribution at single-cell resolution, an effect that is not discovered when only population average measurements are used.

**Fig. 5.—:**
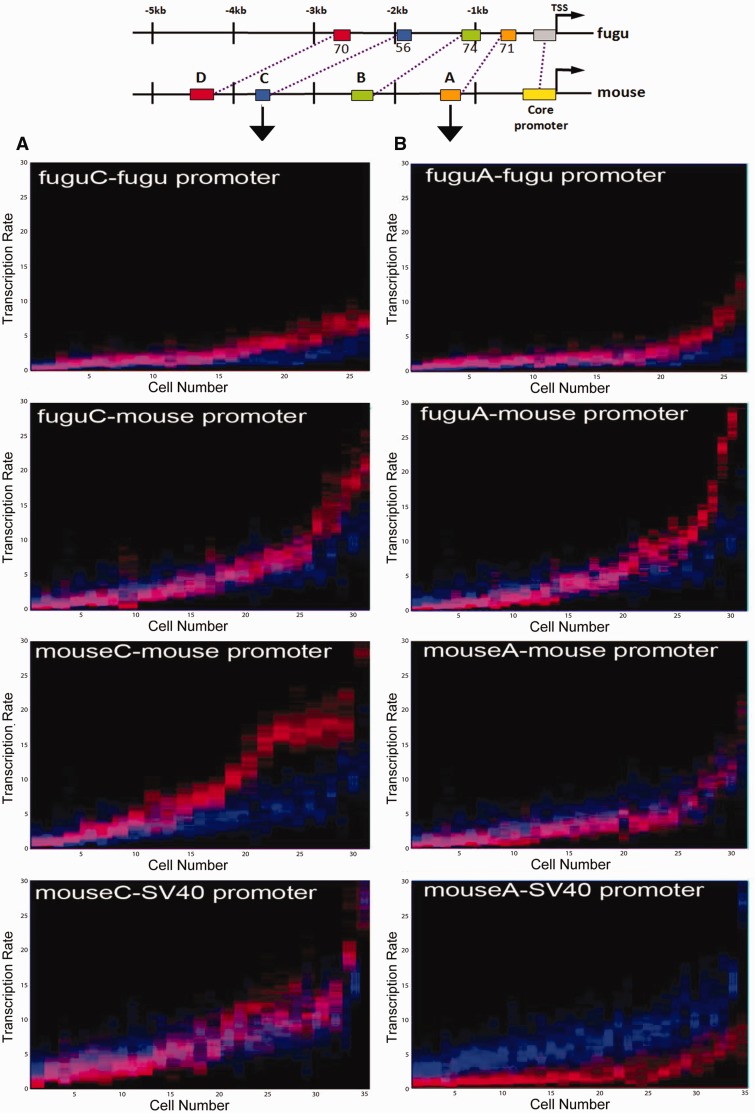
Evolutionary conservation and divergence of *Msx1* CRMC and CRMA function. (*A*) The previously uncharacterized, CRMC from fugu and mouse, acts as an enhancer upon various promoters. The intracellular rate ranges are narrow compared with the basal promoter. (*B*) Sequence conservation does not guarantee functional conservation. Fugu CRMA acts as an enhancer upon the fugu and mouse promoters, whereas mouse CRMA acts as a silencer of the mouse and SV40 promoters. However, despite this evolutionary change in function revealed here, the intracellular SD of transcription rates is very narrow for CRMA and C compared with the basal promoter and CRMB and CRMD containing constructs. Notably, mouse CRMA conveys pure repression upon the SV40 promoter with an extremely narrow intracellular rate distribution.

We next examined the single-cell transcription rate distributions of constructs containing the previously uncharacterized CRMC and CRMA regions. Despite a low sequence conservation (56% between fugu and mouse) CRMC increases the proportion of cells with a higher mean rate, irrespective of the species origin of CRMC or promoter (fig. 5*A* and table 1). However, CRMA, which is highly conserved between fugu and mouse (71%), unexpectedly displays an evolutionary divergence of function as fugu CRMA enhances promoter transcription rates whereas mouse CRMA silences under our identical cellular and experimental conditions. In fact, adding mouse CRMA to SV40 yields a rate distribution below that of the basal SV40 promoter alone (fig. 5*B*), suggesting that sequence evolution within CRMA in the lineage toward mammals has changed its functionality. Surprisingly, the intracellular ranges of rates acquired through adding CRMC and CRMA are consistently narrower than those of the basal promoters, CRMB or CRMD constructs across the entire spectrum of average rates (fig. 5 vs. fig. 4). This is suggestive of a novel CRM intracellular filter function that reduces intracellular variability in transcription rates (compare red and blue in fig. 5*A* and *B* vs. fig. 4*A* and *B*). The use of the posterior probability as a measure of transcriptional variability is discussed in the supplementary text, Supplementary Material online and adds further strength to this result.

### Differential Contributions of Promoters and CRMs to Transcription Rate Distributions in Real Time

**Fig. 6.—:**
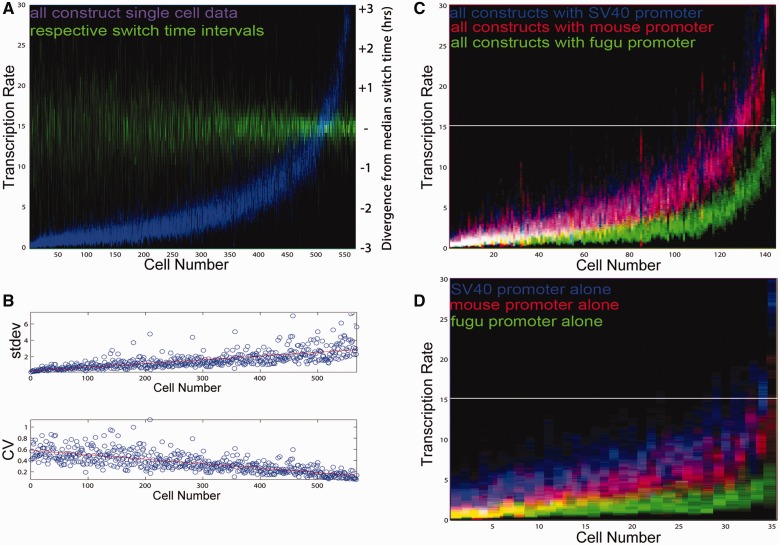
Differential contributions of promoters and CRMs to single-cell transcription rates. Aligning by average rate and color coding all 573 single-cell profiles according to either the promoters or CRMs used allows us to quantify complex promoter dynamics at single-cell resolution. (*A*) The corresponding range of switch times derived from the posterior distribution narrows with increased transcription rate. (*B*) Intracellular SD of estimated rates increases with mean rate (upper graph). At the same time the mean rate increases more quickly than the width of the intracellular rate spectra, leading to a decrease of the intracellular CV (lower graph). (*C*) When color-coded by promoter type, different transcription rate ranges are observed within a given population. Purple is overlap of red and blue; white is overlap of red, green, and blue. Their respective order follows that of the basal promoter rate spectra without CRMs. (*D*) Promoters set specific transcription rate distributions across a population and within each cell.

To perform a statistical analysis on the results as the basis for uncovering general promoter and CRM functional properties, we sorted the transcription rate distributions for all 573 single cells by ascending transcription rate means. This demonstrated that although the range of rates increases with the mean rate inside single cells, the mean grows faster, leading to a CV reduction and therefore an increase in the SNR (=1/CV) (fig. 6*A* and *B*). The results also display a significant contraction of the range of switch time values with increasing single-cell transcription rate means, suggesting that the more rapid transition at higher transcription rates is a global phenomenon (fig. 6*A*). We next color-coded single-cell rate distributions depending on the different promoters used to determine the contributions of promoters to the overall transcription rate distribution (fig. 6*C*). This showed that the rate distributions are separable and reflect those of the underlying basal promoter-alone constructs (fig. 6*D*).

**Fig. 7.—:**
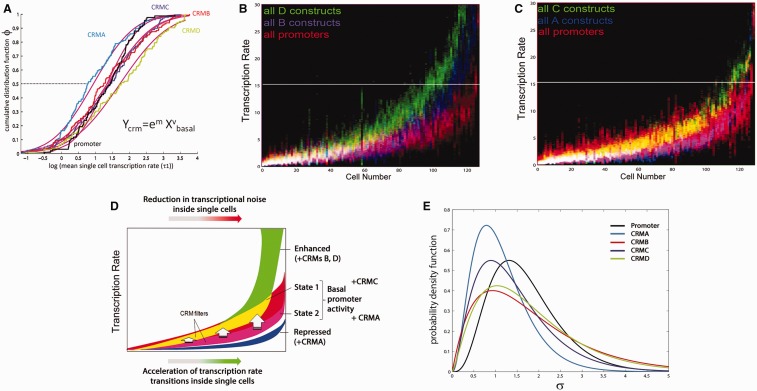
Identification of a novel CRM robustness control function for *Msx1* CRMA and CRMC. (*A*) CRMs affect means and variances of average rates within a population of cells in a nonlinear fashion. A cumulative distribution function of single-cell rate averages in log space provides a generic description for CRM action at the population scale with single-cell resolution. Plotting entire single-cell rate distributions reveals dynamic enhancer functionalities of CRMB and CRMD (*B*) and novel intracellular rate filter functions of CRMA and CRMC (*C*). In these graphs, all single-cell data are color-coded as indicated based on the CRMs they contain. (*D*) Overview graphic displaying the quantitative properties of promoter behavior and CRM action. This highlights two different activity states (yellow and pink) of the basal promoter, each regulated by a different CRM. Given that the spread of rates (SD) increases with increased average rates in shorter periods of time, this implies acceleration of transcription rate choices as a means to accomplish the described distributional properties. (*E*) Distribution of standard deviations for transcription rate estimates indicates repression of transcriptional noise for all CRMs. Gamma distributions were fitted to the standard deviations of the transcription rate MCMC estimates for every cell in each CRM configuration across all promoter types. As all estimations were performed under identical parameterizations, this showed that the uncertainty in the transcription rate estimate for the promoter alone was generally higher across the population, indicating that the time series themselves were more variable due to transcriptional noise.

To investigate the regulatory roles of CRMs, we plotted the averages of all single-cell transcription rates in a log scale as a cumulative distribution function across the population and discovered that a Kolmogorov–Smirnov test finds log-normality for each CRM (purple lines in fig. 7*A*). CRMs lead to a shift in the basal promoter population mean or variance in log space (black in fig. 7*A*). Enhancer CRMs (such as CRMB, C, and D) increase, whereas silencers (CRMA) decrease the basal promoter population mean rate (black stippled line in fig. 7*A*) with each individual CRM modulating the mean with differing strength. CRMB, C, and D cause more cells within a population (at ϕ > 0.5) to acquire a higher average transcription rate (fig. 7*A* and *B*). The continuous nature of such dynamic features would not be visible if only population averages were measured and could be mistaken for ultrasensitive behavior (Hu et al. 2008). Our results therefore show that *Msx1* CRMs function in a graded, nonlinear manner to transform basal promoter rate ranges at single-cell resolution.

### Identification of Conserved CRMs that Function to Reduce Intracellular Variability in Transcription Rates

Our real-time single-cell transcription rate estimations allowed us to investigate the role of CRMs in controlling variability and robustness in *Msx1* expression. The results showed that the intracellular transcription rate ranges for CRMA and CRMC containing constructs are consistently narrower than those of the basal promoters and CRMB or CRMD containing constructs across the entire range of average rates (fig. 7*C* compared with *B*). Despite the fact that CRMA can be either activating (fig. 5*B*, fugu CRMA) or silencing (fig. 5*B*, mouse CRMA), CRMA’s intracellular transcription rates (blue) are largely contained within (pink) the basal promoter (red) single-cell rates and occupy its lower half in the overall cell population. The same is true for CRMC (green), where the overlap (yellow) is confined to the upper bound of the intracellular rate distribution of the basal promoter in 80% of single-cell profiles. This action of cutting out the higher or lower ranges of the intracellular basal promoter rate range represents a novel functionality of CRMs that operates to reduce variability in intracellular transcription rates, irrespective of each cell’s average rate (depicted graphically in fig. 7*D*). Furthermore, distribution of standard deviations for single-cell transcription rate estimates indicates that variations in intracellular transcription rates are reduced for all CRM containing constructs compared with the promoter-alone (fig. 7*E*). This reduction is most pronounced for CRMA and CRMC constructs confirming the capacity of *Msx1* CRMs to reduce intracellular variability in transcription rates. The results therefore suggest that *Msx1* CRMs can function as important regulators of robustness in the control of gene expression in addition to modulating transcription rates.

## Discussion

Random fluctuations in gene expression can be either beneficial or deleterious for a number of fundamental biological processes and can be subject to regulatory control. For example, noise in the expression of key dose-dependent genes must be filtered out to ensure robust cellular function (Newman et al. 2006; Batada and Hurst 2007; Lehner 2008). In this study, we used mathematical modeling to estimate transcription rates for 573 single-cell reporter time courses and analyzed the function of CRMs and promoters in controlling variability and robustness in *Msx1* expression at single-cell resolution.

We assessed the contribution of *Msx1* CRMs to the well-described gene expression level–noise relationship. We found that both the fugu and mouse *Msx1* basal promoters and the heterologous SV40 control promoter displayed a wide range of rates. The shapes of these rate distributions were determined by the species origins of the component used (fig. 6*C* and *D*). We observed a basal promoter-based robustness control that is dependent upon the average rate: The higher the average rate within a given cell, the lower the CV and the higher the SNR (fig. 3*E*). This observation held true for all CRM containing constructs (fig. 6*B*) demonstrating that the noise–mean rate relationship is indeed solely promoter-controlled in vertebrates. These results imply that distal CRMs do not affect the noise–mean expression relationship and are consistent with studies in yeast implicating promoter sequence and nucleosome occupancy at the transcriptional start site in expression noise control (Tirosh and Barkai 2008; Hornung et al. 2012; Carey et al. 2013). In addition to this, recent work in worms has demonstrated promoter-based robustness control in spatial expression and suggested that promoter sequence and length can qualitatively affect promoter robustness (Barriere et al. 2011). Our findings expand on this by analyzing the temporal characteristics of promoter function.

We also investigated the mode of CRM action at single-cell resolution and found conservation of CRM function despite low overall DNA sequence similarity, an observation congruent with the billboard model of CRM function (Arnosti and Kulkarni 2005), as well as divergence of function among highly similar sequences. We discovered that both *Msx1* enhancer and silencer CRMs transform basal promoter rate distributions in a CRM-specific but nonlinear fashion across a population (fig. 7*A*). This suggests that *Msx1* CRMs act to modulate fluctuating promoter rate distributions (fig. 7*E*), and not a single fixed rate as suggested from single time point measurements, in a graded manner. We also show that cell-to-cell variability in transcription rate averages across a population is log-normally distributed (fig. 7*A*), whereas the estimated rates inside a single cell are not, suggesting underlying multiplicative effects acting upon a fundamentally stochastic process within a population in accordance with current thermodynamic models of combinatorial transcription factor binding (Janssens et al. 2006; Segal et al. 2008; Gertz et al. 2009). Although intercellular variability of transcription rate averages is dominated by promoter and species-specific features (figures 6 and 7), different CRMs affect inter- and intracellular rates independently (fig. 7*B* vs. 7*C*). This leads to mean and variance shifts in the log-scale (for CRMB, C, and D) across a population and/or to variance reduction (CRMA, C) in the intracellular transcription rate distributions (fig. 7*A*, *E*).

Previous single molecule studies have shown that transcriptional events are discrete and occur in bursts, interspersed by pauses whose length is proportional to the inverse of the transcription rate (Davenport et al. 2000; Galburt et al. 2009; Hodges et al. 2009). Although the stability of the fluorescent reporter used in our study limited our ability to detect transcriptional bursts, we speculate that distal CRMs may function as individual operating units to regulate either burst size (the number of mRNA molecules produced per burst) or frequency (the rate at which a promoter changes from an inactive to an active state) as shown for promoter proximal transcription factor binding sites (Suter et al. 2011; Hornung et al. 2012; Carey et al. 2013). Indeed, studies in yeast have demonstrated that transcriptional variability can be decoupled from mean expression by differential regulation of either burst frequency or strength (Murphy et al. 2010; To and Maheshri 2010). Future comparative analyses, using destabilized reporters, are thus needed to understand the molecular causes for these dynamic CRM and promoter functions.

In contrast to promoter-based robustness control that is becoming stronger with higher average transcriptional rates within a population, CRM-mediated reduction in intracellular variability covers the entire spectrum of rates within a population (fig. 7*C* and *D*). This indicates that CRMs can function as important regulators of gene expression robustness to ensure that the expression of key developmental transcription factors is precisely controlled. A comparison between fugu and mouse CRMA reveals a change in its function from enhancer to repressor under the same conditions (fig. 5). Despite this change, the filter function of CRMA remains shared across mouse and fugu (fig. 7*C*), suggesting that this is inherited from a common ancestor whereas other rate modulating activities of the same CRMs have evolved separately. Such evolutionary decoupling of rate modulating CRM activities from transcriptional robustness control could provide a mechanism for individual genes to be expressed faster (or slower) than others without affecting their accuracy. We expect that transcription rate filtering is just one in a large class of dynamic functionalities of vertebrate CRMs that remain to be discovered using quantitative single-cell transcription rate measurements.

## Supplementary Material

Supplementary text, figure S1, and table S1 are available at *Genome Biology and Evolution* online (http://www.gbe.oxfordjournals.org/).

Supplementary Data
