# Supplementary material for: Conserved Cis-Regulatory Modules Control Robustness in Msx1 Expression at Single-Cell Resolution
Source: Genome Biol Evol. 2015 Sep 4;7(9):2762–78. doi: 10.1093/gbe/evv179 (PMC4607535; doi:10.1093/gbe/evv179)
Supplement: Supplementary Data [file supp_7_9_2762__index.html]

Conserved Cis-Regulatory Modules Control Robustness in Msx1 Expression at Single-Cell Resolution — Supplementary Data 

# Conserved *Cis*-Regulatory Modules Control Robustness in *Msx1* Expression at Single-Cell Resolution

## Supplementary Data

files

- Supplementary Data - xlsx file
- Supplementary Data - pdf file
- Supplementary Data - pdf file
