# Supplementary material for: Conserved Cis-Regulatory Modules Control Robustness in Msx1 Expression at Single-Cell Resolution
Source: Genome Biol Evol. 2015 Sep 4;7(9):2762–78. doi: 10.1093/gbe/evv179 (PMC4607535; doi:10.1093/gbe/evv179)
Supplement: Supplementary Data [file supp_evv179_Vance_Supplemental_Figure_1.pdf]

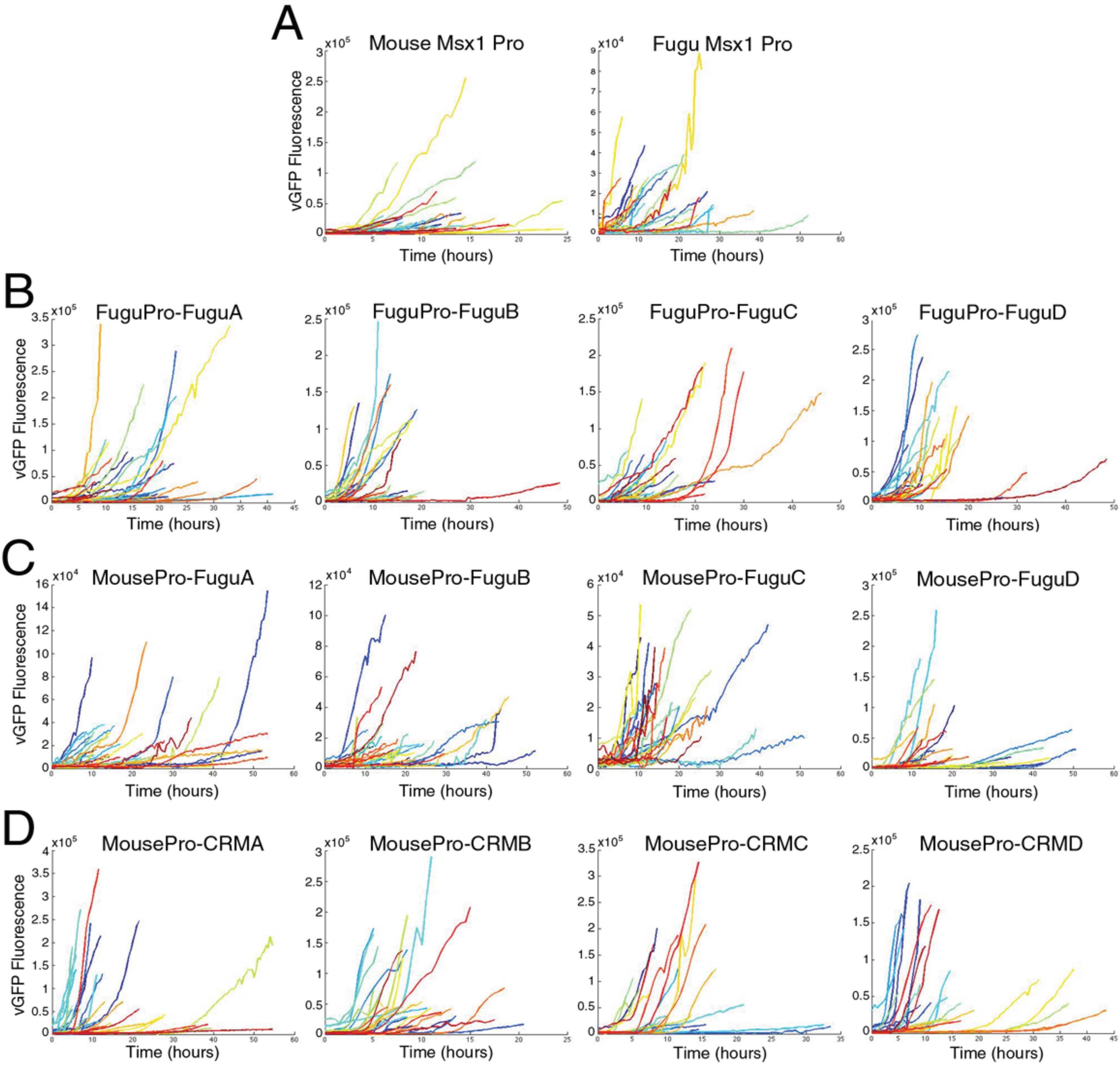

Supplemental Fig. 1. Single cell fluorescent onset curves. Species specific *Msx1* promoter alone (A), fuguCRM-fugu promoter (B), fuguCRM-mouse promoter (C) and mouseCRM-mouse promoter (D) reporter constructs were transfected into Hoechst labelled C2C12 cells. Real time images were acquired at 30 min intervals after transfection. Different colours represent individual fluorescence response curves.
