# Supplementary material for: Conserved Cis-Regulatory Modules Control Robustness in Msx1 Expression at Single-Cell Resolution
Source: Genome Biol Evol. 2015 Sep 4;7(9):2762–78. doi: 10.1093/gbe/evv179 (PMC4607535; doi:10.1093/gbe/evv179)
Supplement: Supplementary Data [file supp_evv179_Vance_Supplemental_Text.pdf]

Conserved *cis*-regulatory modules control robustness in *Msx1* expression at single cell resolution

Keith W. Vance, Dan J. Woodcock, John E. Reid, Till Bretschneider, Sascha Ott and Georgy Koentges

Supplemental Text

Section 1: Supplemental Computational Methods

Section 2: Use of the posterior probability as a measure of transcriptional variability

Section 1: Supplemental Computational Methods

Fugu possesses a single *Msx1* locus

We confirmed by reciprocal BLAST searches of mouse and fugu MSX1 protein sequences against the genomes that there are two scaffolds in the fugu assembly with a potential *Msx1* locus (*Takifugu rubripes*, Ensembl version 57.4k used in this study). Each locus is annotated as an *Msx1* gene in this version of the Ensembl database (gene-IDs ENSTRUG00000002318 and ENSTRUG00000016043). However, one of these loci is on a very small scaffold (scaffold_3613) of only 8,126 nucleotides. The scaffold containing the other locus (scaffold_116, with 924,357 nucleotides) is nearly identical in sequence around the *Msx1* locus, fully containing the sequence of scaffold_3613. The small scaffold is, therefore, redundant as is the annotation of the second *Msx1* locus. As this fugu assembly covers about 98 percent of the fugu genome we conclude that fugu has likely only one copy of *Msx1* left.

Minimal promoter identification usually requires truncation experiments. The mouse minimal *Msx1* promoter has previously been defined experimentally. We therefore surmised that if we can make precise and unbiased predictions of shared transcription factor binding sites (TFBSs) with a high likelihood of being bound by TFs present in cells expressing *Msx1*, this might aid in the *in silico* identification of the orthologous fugu promoter region for further experimentation. To this end we scanned the mouse and fugu sequences of each CRM for putative TFBSs using PSSMs extracted from the TRANSFAC (Matys, et al. 2006) database of TF binding sequence preferences.

Experimental filtering of TRANSFAC TFBS predictions

In order to reduce the complexity of possible TFBSs identified by TRANSFAC without bias we filtered consensus TRANSFAC identifiers by present calls on the basis of microarray experiments of MSX1 positive cells obtained *in vivo* and *in vitro*. Laser capture microdissection was used to generate cDNA of branchial arch neural crest cells (controlled using the *LacZ* mRNA of the *wnt1*-Cre X Rosa26R transgene), they were checked by Southern for *Msx1* cDNA presence according to

our established protocol, described in (Tietjen, et al. 2003). cDNA of MSX1 positive C2C12 cells, our assay system for all further experiments, was also used. cDNAs were analyzed for present/absent calls on Affymetrix microarrays using standard Affymetrix software (expression data not shown) and only those TFs called 'present' were allowed for further analysis. Precise names of present, predicted to be bound TFs with a high score are shown in Figure 1B in the main text.

TFBS Scoring algorithm and fugu promoter identification – BiFa tool

A PSSM of length K induces a distribution over K-mers that models binding sites for the transcription factor(s) it represents. Each position is modeled independently in this distribution. The PSSM can be represented as a matrix, P, where $P_{k,b}$ represents the probability of seeing base b at position k in the PSSM. Given a K-mer, $W = w_1 \ldots w_K$, and using a simple uniform background model, the log odds ratio L(W) between the binding site model (the PSSM) and a uniform 0-order background model is

$$L_P(W) = K\log 4 + \sum_{k=1}^{K} \log P_{k,w_k} + V$$

where V is a prior representing how likely one believes binding to be. $V = -4.7$ (all logarithms to base 10) was used based on experience analyzing other loci. The PSSMs were created using TRANSFAC frequency data smoothed with a pseudo-count of 1. Although many different pseudo-counts are often used in the literature, a survey described in (Nishida, et al. 2009) found 1 to be a suitable value.

Once we had a log-odds ratio for a PSSM binding to each K-mer on both strands of a sequence, we estimated the log-odds ratio that it binds to at least one position in the sequence. For each CRM, we adjusted the log-odds scores for each PSSM on the mouse sequence by averaging them with the log-odds ratio that the PSSM has a TFBS in the CRM's fugu sequence. So PSSMs that have strong sites in the fugu sequence scored more highly in the mouse sequence. It is normal to add log-

odds scores as they measure the weight of evidence in favour of a model, but because our sequences have been selected by conservation, the scores are not independent and adding is not suitable. We have found empirically on other loci that averaging the log-odds scores is a suitable method to integrate phylogenetic information and improve predictions. It is certainly more conservative at predicting binding sites.

Our scoring scheme takes account of phylogenetic conservation of TFBSs in a statistical framework. Additional analyses on top of the scoring scheme retrieved the most significant conserved chains of TFBSs whose order is conserved in the two sequences. For the latter we looked in the analyses for the highest scoring chain of non-overlapping binding sites that was conserved across mouse and fugu. Each chain was scored as the sum of the log-odds ratios for the TFBSs it contained. In this way we compared short conserved chains of strong TFBSs against longer conserved chains of weaker TFBSs. Note order conservation of TFBSs in orthologous fugu and mouse Msx1 promoters in Figure 1B (main text). Using the experimentally defined minimal promoter region in mouse as a reference, we chose that particular part of fugu sequence with the most conserved TFBSs and conserved chains of TFBSs between the two species.

Section 2: Use of the posterior probability as a measure of transcriptional variability

The basis of our parameter estimation utilises Markov Chain Monte-Carlo (MCMC) method, specifically the Metropolis-Hastings algorithm, upon which we build our hierarchical estimation scheme. In this algorithm we consider the parameter of interest as a distribution, and then aim to infer this distribution through the MCMC sampling method, which takes iterative random samples of the parameters and determines their capacity to fit the data using a likelihood function derived from an intermediary model. When the algorithm is run for an appropriate number of iterations (referred to as the Markov chain), the values in the chain can be considered to be equivalent to draws from the distribution of interest, which is known as the posterior distribution and can be reconstructed from these values. We then generally take the mean of this distribution to be the parameter value used in the model.

Aside from checks to determine the algorithm is working correctly, the posterior distribution itself is often discarded, with any variation and structure in the distribution dismissed as uncertainty arising in the data and estimation procedure. However, in the Bayesian paradigm upon which the Metropolis-Hastings algorithm is based, the parameters are treated as random variables rather than fixed parameters. As such, there is no single 'correct' parameter value and instead the values at each iteration in the chain constitute a legitimate parameter set, where the applicability of the value in describing the data is proportional to its frequency in the chain. As such, in Bayesian schemes such as this, imputing the distribution of the parameter values is the objective and so it is natural to infer information from the posterior itself. For example, analysis of the full posterior distributions calculated using MCMC of the switch time parameter has been used to provide evidence for a transcriptional refractory period (Harper, et al. 2011).

In our case, we are estimating parameters corresponding to biological rates using a model of transcription and translation. In this model the parameters are fixed throughout the time course. However, like all models, this is a level of abstraction that we have decided upon

to enable us to best fulfil the goals of the study, under constraints of convenience and tractability, and should not be considered to encapsulate everything within the entire process. In particular, despite our best efforts to incorporate intrinsic noise using the linear noise approximation, and extrinsic noise via the hierarchical distributions, we have not accounted for all sources of stochasticity that may be present in the underlying processes. For example, if we consider the case for the transcription rate[1], as it most pertinent to this study, we know that transcription occurs in bursts, which may be of highly random duration and intensity, may be caused by transcription factors or release from a stalled configuration, and may be affected by innumerable other biological mechanisms. It is impossible to gather data about the relative contributions of all these individual processes, and so we model them all by a single parameter, in this case τ_i. As there are many such factors that can affect τ_i changing throughout the time course, a fixed parameter will be only represent a composite of these processes. However, from the Bayesian perspective where there is no definitive value of τ_i, only the distribution over legitimate values, it is reasonable to assume that changes in the underlying sources of stochasticity could lead to changes in the data that themselves lead to different ranges of values that adequately fit the data, even if the mean remains unchanged[2]. As such, we can use the posterior distribution derived from the Markov chains to gain information about differences in the underlying stochasticity. It should be noted that the large number of unknown factors acting on these distributions make meaningful quantification difficult, but it is valid to draw qualitative comparisons between MCMC distribution estimates derived from sets of genetically identical cells such as ours.

[1] similar arguments can be made for all parameters in our model

[2] One could conceptualise this as the unknown sources of stochasticity serve to contribute to our uncertainty of the true value of τ_i, but this is antithetical to the Bayesian approach.

As a proof of principle, we considered a lower layer of abstraction of transcription than our current model, in which transcriptional bursting occurs due to a random telegraph process (Harper, et al. 2011; Paulsson 2005),

$$\gamma_{off} \underset{k_-}{\overset{k_+}{\rightleftharpoons}} \gamma_{on}$$

Here transcription randomly flips from an inactive to an active state with rate k_+, and from an active state to the inactive state with rate k_-. When in the active state, the gene is being transcribed with some transcription rate γ_{on}, and when inactive the rate is γ_{off}. It should be noted that these states differ from those in the main paper which refer to two sequential regimes of different transcriptional activity, this is a model of transcriptional bursting which yields the overall transcription rate in each of those regimes. We created data sets using this process in a Gillespie algorithm (Gillespie 1977), where both transcriptional regimes were subject to this process. We then used our estimation scheme with the fixed parameters as before, and explored the effect of different values of k_+ and k_- affected the resulting distribution estimates of τ_1.

We created three synthetic data sets, in all of which we set the transcription in the inactive state $\gamma_{off} = 0$. In Dataset 1 we put $k_- = 0$, and $\gamma_{on} = 5$, yielding a constant transcription rate $\tau_1 = 5$. For the other data sets, both rate parameters were set to be equal so that the cells would spend an approximately equal amount of time in both states. As such we set $\gamma_{on} = 10$ so that τ_1 would also be equivalent to 5 for comparative purposes. In Dataset 2 we set k_+ and k_- to 5, and in Dataset 3 we set k_+ and k_- equal to 1, resulting in less frequent flipping between states, with the ramification that the duration spent in each state is generally longer and more variable. This is exemplified in Supplemental Figure S1 which shows sample

transcriptional activity profiles taken from each data set and the resulting protein time series generated through the Gillespie algorithm.

Supplemental Figure S2 shows the histograms of the standard deviation values of τ_1 in each of the individual time series for each data set. As we can see, the added stochasticity introduced by the random flipping between the genes manifests in the posterior distributions derived from the MCMC algorithm as greater variation. Furthermore, we note the distribution estimate for τ_1 in Dataset 3 has a greater standard deviation on average than that Dataset 2. This is because Dataset 3 is more susceptible to stochastic fluctuations as the duration of the transcriptional bursts are more variable, meaning that the flipping is more likely to be seen in the resulting time series.

From these results, and previous work in (Harper, et al. 2011), we can be confident that variation in stochastic processes underlying our fixed model parameters is likely to manifest in the estimates of the posterior distributions when using our algorithm.

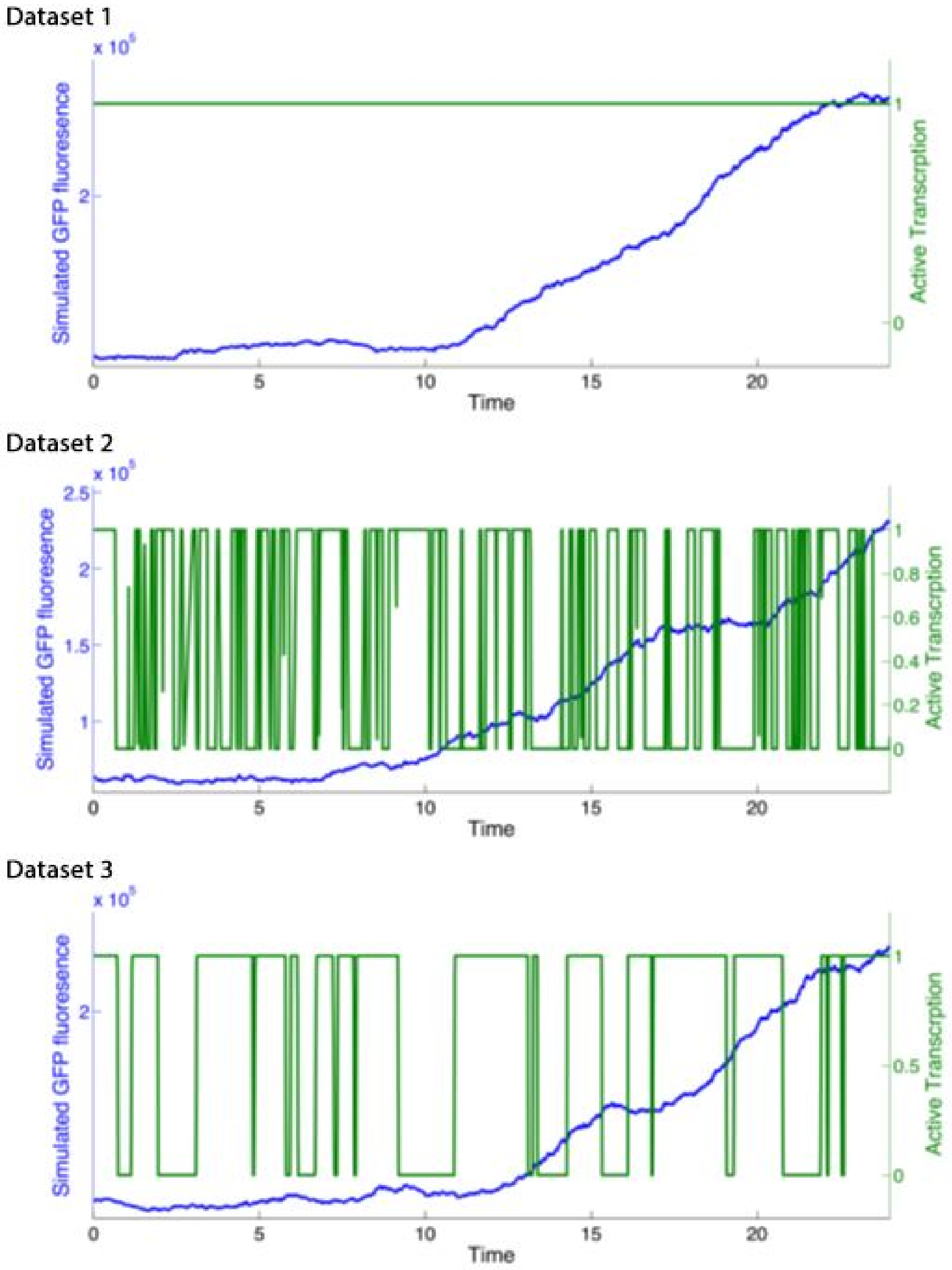

Supplemental Figure S1. Transcriptional activity profiles taken from each data set and the resulting protein time series generated through the Gillespie algorithm.

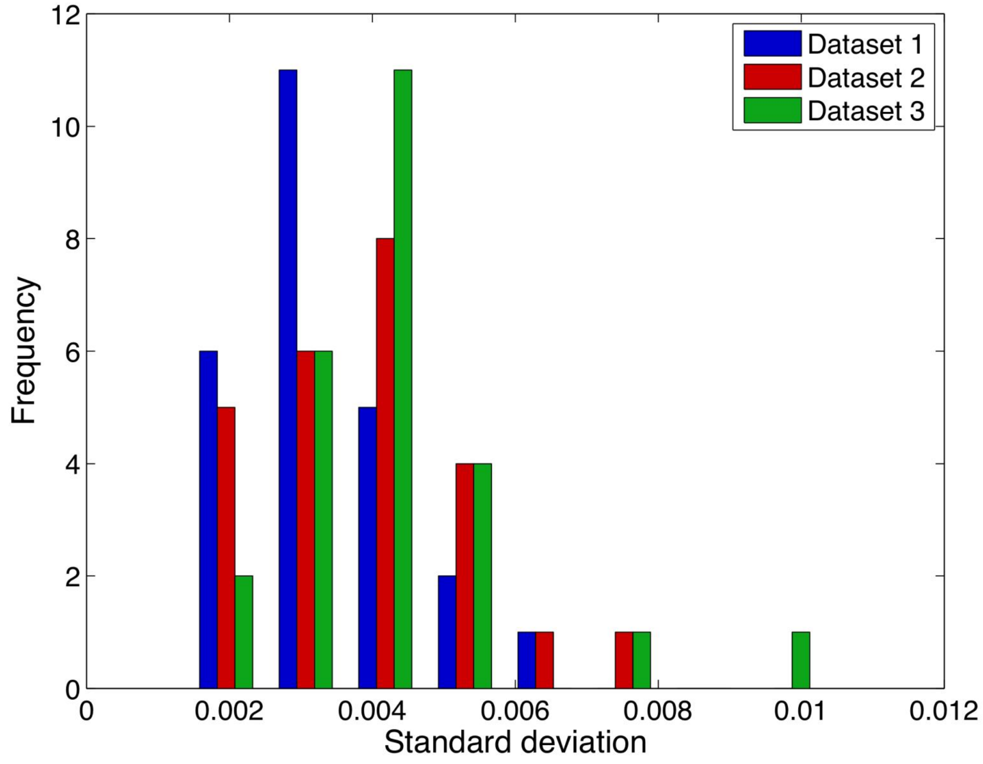

Supplemental Figure S2. Histograms plotting the standard deviation values of τ_1 in each of the individual time series for each data set

References

Gillespie DT 1977. Exact Stochastic Simulation of Coupled Chemical Reactions. The Journal of Physical Chemistry 81: 2340–2361.

Harper CV, et al. 2011. Dynamic analysis of stochastic transcription cycles. PLoS Biol 9: e1000607. doi: 10.1371/journal.pbio.1000607

Matys V, et al. 2006. TRANSFAC and its module TRANSCompel: transcriptional gene regulation in eukaryotes. Nucleic Acids Res 34: D108-110. doi: 10.1093/nar/gkj143

Nishida K, Frith MC, Nakai K 2009. Pseudocounts for transcription factor binding sites. Nucleic Acids Res 37: 939-944. doi: 10.1093/nar/gkn1019

Paulsson J 2005. Models of stochastic gene expression Physics of Life Reviews 2: 18.

Tietjen I, et al. 2003. Single-cell transcriptional analysis of neuronal progenitors. Neuron 38: 161-175.
